# Minimally invasive pulmonary valve replacement via left anterior mini-thoracotomy using an automated fastener-assisted suturing technique: a case report

**DOI:** 10.1093/jscr/rjag469

**Published:** 2026-06-19

**Authors:** Jeong A Lee, Daisuke Takeyoshi, Tasuku Kawarabayashi, Akito Inoue, Kaname Shimizu, Kentaro Shirakura, Yuki Setogawa, Hiroyuki Miyamoto, Ryohei Ushioda, Shogo Takahashi, Shingo Kunioka, Hiroyuki Kamiya

**Affiliations:** Department of Cardiac Surgery, Asahikawa Medical University, Midorigaoka 1-1-1, Asahikawa, Hokkaido 078-8510, Japan; Department of Cardiac Surgery, Asahikawa Medical University, Midorigaoka 1-1-1, Asahikawa, Hokkaido 078-8510, Japan; Department of Cardiac Surgery, Asahikawa Medical University, Midorigaoka 1-1-1, Asahikawa, Hokkaido 078-8510, Japan; Department of Cardiac Surgery, Asahikawa Medical University, Midorigaoka 1-1-1, Asahikawa, Hokkaido 078-8510, Japan; Department of Cardiac Surgery, Asahikawa Medical University, Midorigaoka 1-1-1, Asahikawa, Hokkaido 078-8510, Japan; Department of Cardiac Surgery, Asahikawa Medical University, Midorigaoka 1-1-1, Asahikawa, Hokkaido 078-8510, Japan; Department of Cardiac Surgery, Asahikawa Medical University, Midorigaoka 1-1-1, Asahikawa, Hokkaido 078-8510, Japan; Department of Cardiac Surgery, Asahikawa Medical University, Midorigaoka 1-1-1, Asahikawa, Hokkaido 078-8510, Japan; Department of Cardiac Surgery, Asahikawa Medical University, Midorigaoka 1-1-1, Asahikawa, Hokkaido 078-8510, Japan; Department of Cardiac Surgery, Asahikawa Medical University, Midorigaoka 1-1-1, Asahikawa, Hokkaido 078-8510, Japan; Department of Cardiac Surgery, Asahikawa Medical University, Midorigaoka 1-1-1, Asahikawa, Hokkaido 078-8510, Japan; Department of Cardiac Surgery, Asahikawa Medical University, Midorigaoka 1-1-1, Asahikawa, Hokkaido 078-8510, Japan

**Keywords:** pulmonary regurgitation, adult congenital heart disease, minimally invasive cardiac surgery, pulmonary valve replacement, automated fastener

## Abstract

Chronic pulmonary regurgitation is a late complication after repair of congenital heart disease involving the right ventricular outflow tract. In adult congenital heart disease patients, redo sternotomy carries a risk of cardiac injury due to dense retrosternal adhesions, and minimally invasive approaches have emerged as alternatives. In addition to the surgical approach, prosthetic valve implantation at the posterior pulmonary annulus is technically challenging because knot tying above the valve is difficult in a restricted operative field. We report a 40-year-old man with congenital pulmonary artery dilatation after childhood ventricular septal defect repair who underwent pulmonary valve replacement via left anterior mini-thoracotomy. A 27-mm bioprosthetic valve was implanted in a supra-annular position using interrupted mattress sutures, with an automated knot fastener (Cor-Knot) applied to the posterior annulus. This case demonstrates that minimally invasive pulmonary valve replacement is feasible and reproducible, and that automated fasteners facilitate standard suturing at the posterior pulmonary annulus.

## Introduction

Advances in surgical management of congenital heart disease have led to a growing population of adults requiring late reinterventions. Chronic pulmonary regurgitation (PR) is a late complication following repair of right ventricular outflow tract (RVOT) lesions and may lead to progressive right ventricular dilation and dysfunction if untreated [1, 2].

Although repeat median sternotomy remains the standard approach for pulmonary valve replacement (PVR), redo sternotomy in patients with adult congenital heart disease carries a risk of cardiac injury due to dense retrosternal adhesions [3]. Minimally invasive pulmonary valve replacement (MICS-PVR) via left mini-thoracotomy has therefore been proposed as an alternative that avoids hazardous sternal re-entry and reduces mediastinal dissection [4–7].

However, fixation of the prosthetic valve to the posterior pulmonary annuls can be technically challenging via left mini-thoracotomy. In a median sternotomy approach, the prosthetic valve can be observed and knot tying can be done from the cranial side, but it can be visualized only from the caudal side via left mini-thoracotomy and therefore knot tying can be complex. As a result, alternative techniques such as running sutures or modified interrupted sutures have been employed; however, concerns remain regarding fixation strength and the risk of paravalvular leakage. Tatewaki *et al*. [4] described a thoracotomy approach in which interrupted sutures were tied beneath the prosthetic valve to overcome these limitations.

We report a case of severe PR associated with congenital pulmonary artery dilatation after childhood VSD repair, successfully treated with left anterior mini-thoracotomy PVR. In this case, the prosthetic valve was implanted in a supra-annular position using interrupted pledgeted mattress sutures. An automated knot fastener (Cor-Knot; LSI Solutions, Victor, NY, USA) was selectively applied to the posterior annulus, enabling secure knot fixation above the prosthetic valve without deep manual knot tying.

## Case presentation

A 40-year-old man with a history of childhood VSD repair and congenital pulmonary artery dilatation was referred for evaluation of progressive right ventricular dilatation. Transthoracic echocardiography showed severe PR with preserved left ventricular function (ejection fraction 60%). Cardiac magnetic resonance imaging demonstrated a PR fraction of 47%, right ventricular end-diastolic volume index of 245 mL/m^2^, and right ventricular ejection fraction of 33%. No residual ventricular septal defect was identified. Preoperative computed tomography (CT) demonstrated dense retrosternal adhesions (Fig. 1). Because no intracardiac repair was required and redo sternotomy was considered hazardous, MICS-PVR was selected.

**Figure 1:**
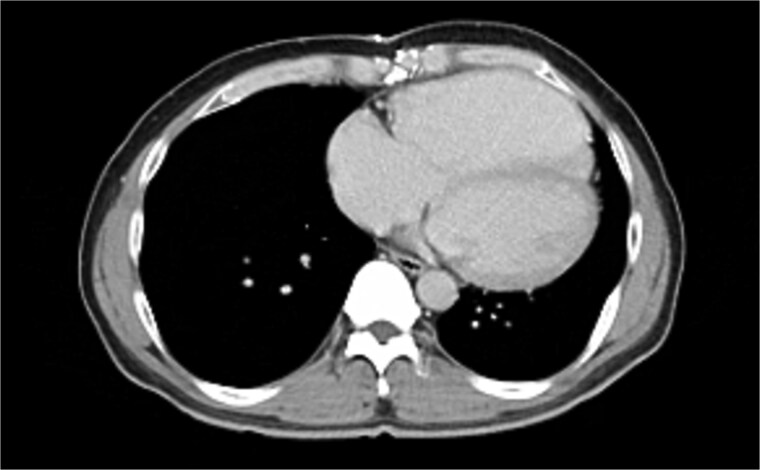
A preoperative contrast-enhanced CT scan confirmed that the right ventricle was located directly under the sternum, raising concerns about the risk of a repeat median sternotomy.

Cardiopulmonary bypass was instituted through femoral arterial and venous cannulation. In the absence of intracardiac shunts, the operation was performed on-pump under beating-heart conditions. A left anterior mini-thoracotomy was performed through the third intercostal space. To improve exposure, partial resection of the left fourth rib was added as needed (Fig. 2a). After opening the pericardium, the RVOT and main pulmonary artery were exposed under direct vision. The RVOT and main pulmonary artery were longitudinally incised, and the pulmonary valve leaflets were excised with removal of residual thickened tissue. A 27-mm bioprosthetic valve was implanted in a supra-annular position using interrupted pledgeted 2–0 braided mattress sutures placed circumferentially from the pulmonary annulus to the sewing cuff. Because knot tying at the posterior annulus is technically demanding in a deep and restricted operative field, most sutures in this region were secured using Cor-Knot, allowing knot fixation above the prosthetic valve without deep manual tying (Fig. 2b). Sutures at the anterior aspect, where visualization was adequate, were tied manually. Following valve implantation, the RVOT was reconstructed using a tailored patch fashioned from a vascular graft, which was sutured with a continuous polypropylene suture to enlarge the outflow tract (Fig. 2c). Hemostasis was confirmed, and the patient was weaned from cardiopulmonary bypass without difficulty. The postoperative course was uneventful.

**Figure 2:**
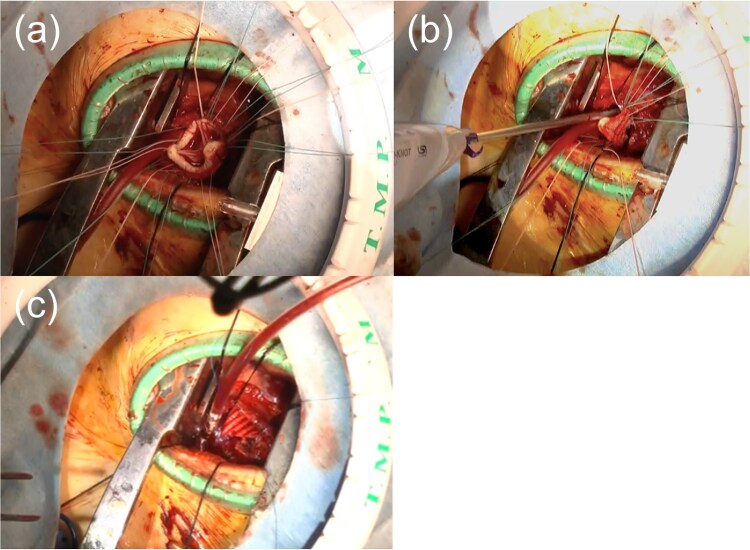
Intraoperative findings. (a) Adequate exposure was achieved via a left third intercostal approach with partial resection of the fourth rib. (b) The prosthetic valve was implanted using interrupted mattress sutures, with a Cor-knot device applied to the posterior annulus. (c) The right ventricular outflow tract was reconstructed using a patch.

## Discussion

This case demonstrates two key points. First, MICS-PVR via left anterior mini-thoracotomy is a feasible and reproducible surgical approach. Second, the use of Cor-Knot facilitates knot placement above the prosthetic valve at the posterior pulmonary annulus.

Redo sternotomy in adult congenital heart disease patients carries a risk of cardiac injury due to dense retrosternal adhesions [3]. Tatewaki *et al*. [4] reported PVR via thoracotomy as an alternative to resternotomy, demonstrating that this approach allows limited mediastinal dissection and may reduce operative bleeding. Minimally invasive thoracotomy approaches have subsequently been reported as feasible options in selected patients [6, 7]. Furthermore, in cases without an intracardiac shunt, PVR does not require aortic cross-clamping and is therefore particularly well-suited to minimally invasive approaches. In the present case, preoperative CT suggested dense adhesions behind the sternum, and no intracardiac repair was required. Therefore, PVR via left anterior mini-thoracotomy was selected to avoid hazardous sternal re-entry, providing a minimally invasive and reproducible strategy.

A key technical challenge in PVR is suturing at the posterior pulmonary annulus. Because the operative field in this region is deep and the working space is limited, knot tying above the prosthetic valve is technically demanding. Running sutures are often used to avoid multiple deep knot placements; however, they may result in less controlled annular fixation, raising concerns regarding uneven tension distribution and the risk of paravalvular leakage. Standard PVR emphasizes secure annular fixation using interrupted mattress sutures [5]. Tatewaki *et al*. [4] addressed this limitation by placing sutures beneath the prosthetic valve, although this requires more complex suture management. In the present case, the prosthetic valve was implanted in a supra-annular position using interrupted mattress sutures. Cor-Knot was selectively applied to the posterior annulus, enabling secure and consistent knot fixation above the prosthetic valve without deep manual knot tying. This approach allows surgeons to maintain a standard suturing technique while overcoming the technical limitations of posterior annular suturing in MICS-PVR.

## References

[1] Geva T . Indications and timing of pulmonary valve replacement after tetralogy of Fallot repair. Circulation 2013;128:1855–7. 10.1161/CIRCULATIONAHA.113.005878 24065609 PMC3898939

[2] Therrien J, Provost Y, Merchant N et al. Optimal timing for pulmonary valve replacement in adults after tetralogy of Fallot repair. Am J Cardiol 2005;95:779–82. 10.1016/j.amjcard.2004.11.037 15757612

[3] Roselli EE, Pettersson GB, Blackstone EH et al. Adverse events during reoperative cardiac surgery: frequency, characterization, and rescue. J Thorac Cardiovasc Surg 2008;135:316–23.e6. 10.1016/j.jtcvs.2007.08.060 18242260

[4] Tatewaki H, Sakamoto I, Ushijima T et al. Pulmonary valve replacement via left thoracotomy as an alternative to resternotomy. Ann Thorac Surg 2020;110:e537–9. 10.1016/j.athoracsur.2020.03.130 32454022

[5] Kotani Y, Kasahara S. Bioprosthetic pulmonary valve replacement. Oper Tech Thorac Cardiovasc Surg 2022;25:13–26. 10.1053/j.optechstcvs.2020.01.001

[6] Carr K, Nijres BM, Windsor J et al. Single-center experience of hybrid pulmonary valve replacement using left anterior thoractomy with pulmonary artery plication in patients with large right ventricular outflow tract. J Am Heart Assoc 2022;11:e024611. 10.1161/JAHA.122.026517 PMC9707832 35861815

[7] Le Ho Y, Khamis AY, Kareem AA et al. Left anterior mini-thoracotomy: an alternative approach for pulmonary valve replacement after surgically corrected tetralogy of Fallot. J Cardiothorac Surg 2024;19:427. 10.1186/s13019-024-02817-5 38982433 PMC11234653

